# Oxidative Stress Biomarkers in Laryngeal Squamous Cell Carcinoma and Their Clinical Implications: Preliminary Results

**DOI:** 10.3390/biomedicines13030667

**Published:** 2025-03-08

**Authors:** Barbara Verro, Carmelo Saraniti, Diana Di Liberto, Giovanni Pratelli, Marianna Lauricella, Daniela Carlisi

**Affiliations:** 1 Division of Otorhinolaryngology, Department of Biomedicine, Neuroscience and Advanced Diagnostic, University of Palermo, 90127 Palermo, Italy; carmelo.saraniti@unipa.it; 2 Section of Biochemistry, Department of Biomedicine, Neuroscience and Advanced Diagnostic, University of Palermo, 90127 Palermo, Italy; diana.diliberto@unipa.it (D.D.L.); giovanni.pratelli@unipa.it (G.P.); marianna.lauricella@unipa.it (M.L.); daniela.carlisi@unipa.it (D.C.)

**Keywords:** laryngeal cancer, reactive oxygen species, oxidative stress, heme-oxygenase 1, metallothionein, superoxide dismutase, catalase, vimentin, nuclear factor erythroid 2-related factor 2

## Abstract

**Background/Objectives**: Laryngeal squamous cell carcinoma represents one of the most common head and neck cancers with a five-year survival rate that, despite diagnostic and therapeutic advances, has not shown any significant improvement in recent decades. Oxidative stress, generated by an imbalance between reactive oxygen species and cellular antioxidant systems, is considered a central mechanism in the carcinogenesis of laryngeal squamous cell carcinoma, causing DNA damage and genomic alterations. **Methods**: This prospective observational paired case–control study focused on the evaluation of antioxidant proteins, such as superoxide dismutase, catalase, heme-oxygenase 1, vimentin, metallothionein, and nuclear factor erythroid 2-related factor 2, in cancer tissues from fifteen patients with laryngeal squamous cell carcinoma, using adjacent healthy tissues as controls. **Results**: The results show a statistically significant overexpression of all proteins analyzed in cancer tissues compared to controls, with relevant correlations between specific biomarkers and clinical characteristics, age, sex, smoking habits, and degree of tumor differentiation. **Conclusions**: These preliminary studies, while limited by sample size and the complexity of molecular regulation, indicate that the overexpression of antioxidant enzymes in laryngeal squamous cell carcinoma tissues, along with their correlations with key clinical parameters, underscores a context-dependent role of oxidative stress in tumor progression. A deeper understanding of oxidative stress mechanisms could contribute to advance personalized management strategies for laryngeal squamous cell carcinoma, potentially improving treatment outcomes and patient prognosis.

## 1. Introduction

Globally, head and neck carcinoma (HNC) accounted for approximately 932,000 new cases and 467,000 deaths worldwide, representing about 3.9% of all cancer cases [1]. Laryngeal squamous cell carcinoma (LSCC) represents one third of all head and neck carcinomas worldwide, with 12,380 new cases in the United States in 2023 [2,3,4]. In the US, the scientific literature has shown a steady decline in laryngeal cancer incidence and mortality rates [2,5]. LSCC more often affects men, with a M:F ratio of 4:1, largely because cigarette smoking—the primary risk factor for LSCC—is more prevalent among males [6,7]. Laryngeal cancer is closely linked to risk factors such as tobacco and alcohol consumption. Smoking is the main cause: smokers are 10 to 15 times more likely to develop this type of cancer than non-smokers, while heavy smokers can have up to 30 times more risk [8,9]. Alcohol consumption is another important risk factor for LSCC, with numerous studies confirming this association. Several studies demonstrated that the risk of developing this cancer increases proportionally to alcohol consumption: for every 10 g of alcohol per day, the risk increases by 9%, with significant risk in moderate and heavy drinkers [10,11,12]. Exposure to different environmental and occupational factors, such as asbestos, textile dust, and polycyclic aromatic hydrocarbons, is thought to potentially increase the risk of LSCC [13,14]. In addition, the possible involvement of laryngopharyngeal reflux (LPR) in the occurrence of the disease is still being discussed and studied [15]. Also, food may play a role in the development of LSCC: in particular, the intake of a greater variety of vegetables and fruits is associated with a significantly reduced risk of laryngeal cancer, with the risk being lowered by about 60% compared to those who consume fewer vegetables and fruits. In contrast, a higher intake of meat is associated with a higher risk of LSCC, with an increase of 67% [16].

Thus, LSCC pathogenesis is not yet fully understood, requiring further investigation. The main hypothesis suggests that oxidative stress plays a role in causing DNA damage by overwhelming antioxidant enzyme activity [17]. Several studies have shown that reactive oxygen species (ROS) play a crucial role in various aspects of malignancy, including tumor development and abnormal growth, metastasis, and angiogenesis [18,19]. ROS are chemically reactive oxygen-containing molecules produced by both the mitochondria and external factors such as ionizing radiation. Normally, the production of ROS and cellular antioxidant mechanisms are in dynamic equilibrium. However, when ROS levels exceed the cell’s antioxidant capacity, an oxidative stress state occurs. This stress can alter the activity of antioxidant enzymes and damage DNA, leading to genomic instability. The damage mediated by ROS to cellular macromolecules is closely associated with carcinogenesis processes [17,20]. Antioxidant enzymes protect cells from free radical damage. At the same time, the further increase in ROS levels leads to toxic effects that could promote cancer cells death [20,21,22,23,24].

Despite the introduction of new diagnostic techniques, ongoing pathogenesis studies, and new therapeutic solutions, LSCC remains one of the oncological diseases in which the 5-year survival rate has not decreased significantly over the last 40 years, from 66% to 61%, although the overall incidence of the disease has decreased [2,25]. Local relapses and the onset of distant metastases, which often do not respond to conventional treatments, are among the possible causes of this unchanging trend [26]. These data underline the urgency and need of identifying new reliable prognostic biomarkers to improve management of this disease.

Based on this assumption, the present study focused on the analysis of the expression of antioxidative proteins in samples of laryngeal tissue, comparing LSCC cancer tissues with surrounding healthy tissues.

In particular, we focused on six key oxidative stress-related proteins: superoxide dismutase (SOD), catalase (CAT), heme oxygenase-1 (HO-1), nuclear factor erythroid 2-related factor 2 (NRF-2), metallothionein (MT), and vimentin (VIM). These proteins were selected based on their well-established role in redox homeostasis, inflammation, and tumor progression. SOD and CAT are primary antioxidant enzymes responsible for detoxifying superoxide radicals and hydrogen peroxide, respectively [27,28]. HO-1 and NRF-2 play central roles in cytoprotective responses to oxidative stress [29,30], while MT contributes to metal ion homeostasis and free radical scavenging [31]. Finally, VIM, despite being traditionally known as a cytoskeletal protein, has been implicated in epithelial-to-mesenchymal transition (EMT) and oxidative stress regulation [32]. Understanding the expression of these proteins in LSCC may provide insights into oxidative stress-driven tumorigenesis and potential prognostic biomarkers.

## 2. Materials and Methods

The research project is a prospective observational paired case–control study carried out on patients affected by LSCC who came to the Otolaryngology Unit of the University Hospital Paolo Giaccone of Palermo from 1 November 2023. The study received approval from the Ethics Committee of our University Hospital (protocol no. 05/2023), and all enrolled patients provided informed consent to participate in accordance with the Declaration of Helsinki. Only patients who met the following inclusion criteria were recruited for the study: (1) patients over 18 years old, (2) males and females, (3) histopathological diagnosis of LSCC, and (4) any TNM stage of LSCC. Patients were excluded from the project in cases of (1) previous RT and/or chemotherapy and/or immunotherapy in the head and neck, (2) contraindications to surgery under general anesthesia, (3) histological diagnosis different from SCC, (4) multiple primary cancers, (5) patients lacking decision-making capacity, and (6) pregnancy.

### 2.1. Clinical Evaluation

#### 2.1.1. Patients’ Data Collection

Once the patients were selected, demographic data (sex, age, occupation), behavioral use (smoking, alcohol), and comorbidities (e.g., diabetes mellitus, LPR, previous cancers) were collected. Regarding the “tobacco use” parameter, the patients were divided into non-smokers, light smokers (less than 20 cigarettes per day), and heavy smokers (more than 20 cigarettes per day). This cutoff is both clinically and behaviorally relevant, as it corresponds to the typical contents of a standard cigarette pack. This makes the classification intuitive and practical, as it reflects a natural distinction in smoker behavior. Regarding the parameter of “alcohol consumption”, the population was classified into non-drinkers and drinkers of less or more than 4 drinks per day, where any drink, whatever the type, contains about 14 g of ethanol [33]. This approach reduces variability and potential confusion that can arise from differing definitions of ‘standard units’ across countries and studies, ensuring consistency and comparability in our findings.

The tumor features analyzed were as follows: site (supraglottic, glottic, subglottic, transglottic, and pharyngo-laryngeal), degree of differentiation (G1 or well-differentiated, G2 or moderately differentiated, G3 or poorly differentiated), clinical TNM and/or pathological TNM (Tumor-Nodes-Metastasis) according to TNM Classification of Malignant Tumors of the American Joint Committee on Cancer (AJCC) (8th edition) [34], possible Human Papilloma Virus (HPV) expression in laryngeal sample, Programmed Death-1 Ligand 1 (PD-L1) expression level in laryngeal sample, and C-reactive protein (CRP) expression level in serum (cut-off < 5 mg/L). The CRP was measured at a single time, immediately before surgery, when the patient did not present any active inflammatory or infectious processes. This preoperative timing was chosen to avoid results that were confused by the inflammatory response induced by the surgical procedure itself. Preoperative assessment has allowed for a more accurate detection of the patients’ basal inflammatory status.

HPV testing was performed by using the +p16 IHC as a Surrogate for Transcriptionally Active High-Risk HPV: negative when less than 50% diffuse and moderate-to-strong nuclear and cytoplasmic staining; equivocal if less than 70% but greater than 50% diffuse and moderate-to-strong nuclear and cytoplasmic staining; positive if greater than or equal to 70% diffuse and moderate-to-strong nuclear and cytoplasmic staining [35]. Immunohistochemical characterization of PD-L1 on LSCC was performed by using Dako PD-L1 IHC 73-10 assay (Agilent, Santa Clara, CA, USA) and the expression level was scored based on a Combined Positive Score (CPS): CPS < 1; CPS ≥ 1 which means eligibility for first-line treatment with pembrolizumab, and CPS ≥ 20, which does not determine eligibility for first-line treatment with pembrolizumab as a single agent [36]. Also, the treatment was recorded: type of surgery (transoral laser, partial or total laryngectomy with or without neck dissection) and/or radiotherapy (RT) and/or chemotherapy (CT), either alone or as adjuvant to surgery.

#### 2.1.2. Samples Harvesting

Once the patients were selected, the laryngeal specimens were harvested. Under general anesthesia, during transoral or open laryngeal surgery, both the LSCC specimen (case) and the adjacent healthy laryngeal tissue (control) were collected from the same patient to minimize interindividual variability bias. The collected tissue samples, from both the tumor (case) and the adjacent healthy laryngeal (control) tissues, were bisected; one half of each specimen was submitted for histopathological examination to confirm that the adjacent tissue used as control was indeed histologically normal and free of pathological changes. To avoid bias related to the variability of different sites of laryngeal epithelium, samples were taken from the same anatomical region of the larynx. Dry samples have been stored frozen at −20 °C for a limited period before transfer to −80 °C.

### 2.2. Biochemical Evaluation

#### 2.2.1. Protein Extraction from Tissue Samples

Aliquots of the frozen tissues (0.02 to 0.10 g) were washed multiple times with phosphate-buffered saline (PBS) and homogenized in an ice bath using a Radioimmunoprecipitation assay (RIPA) buffer (1 × PBS, 1% NP40, 0.1% SDS (Sodium dodecyl sulfate), 5 mmol/L EDTA (Ethylenediaminetetraacetic Acid), 0.5% sodium deoxycholate) supplemented with protease and phosphatase inhibitors (0.01% aprotinin, 10 mM sodium pyrophosphate, 2 mM sodium orthovanadate, 1 mM PMSF (phenylmethylsulfonyl fluoride). The cellular lysate was centrifuged to remove debris and stored at −80 °C.

#### 2.2.2. Western Blot

After sonication (Soniprep 150, Cellai SRL, Milan, Italy), protein concentrations were determined using the Bradford Protein Assay (BioRad, Hercules, CA, USA). The samples (30 µg of proteins) were inactivated at 95 °C for 5 min, separated by SDS-PAGE, and transferred onto nitrocellulose membranes by electroblotting. Immunodetection was performed using primary antibodies directed against superoxide dismutase (SOD, 25 kDa, purchased from Santa Cruz Biotechnology, Dallas, TX, USA), catalase (CAT, 60 kDa, purchased from Sigma-Aldrich, St. Louis, MO, USA), heme-oxygenase 1 (HO-1, 33 kDa, purchased from Biorbyt, Cambridge, UK), metallothionein (MT, 14 kDa, purchased from GeneTex, Alton Pkwy Irvine, CA, USA), vimentin (VIM, 50 kDa, purchased from Santa Cruz Biotechnology, Dallas, TX, USA), and nuclear factor erythroid 2-related factor 2 (NRF2, 61 kDa, purchased from Novus Biologicals, Centennial, CO, USA), incubated overnight at 4 °C. The membranes were then incubated with specific HRP-conjugated secondary antibodies (purchased from Promega, Milan, Italy) for 1 h at Room Temperature (RT). The signal was detected by enhanced chemiluminescence (ECL) and quantified using CHEMIDOC (BioRad Laboratories, Hercules, CA, USA) and densitometric analysis (Quantity One software, version 4.6.6), normalized to the house-keeping protein mucin-1 (MUC-1, 122 kDa, purchased from Santa Cruz Biotechnology, Dallas, TX, USA). All experiments were conducted in triplicate.

### 2.3. Statistical Analysis

Descriptive statistics were reported as numbers and percentages or mean ± standard deviation (SD). Results were represented as histograms with error bars (Standard Error of the Mean, SEM), and statistical significance was indicated by asterisks (* *p* < 0.05; ** *p* < 0.01; *** *p* < 0.001). Paired *t*-tests were performed to assess the differences between tumor and adjacent healthy tissues. Pearson’s correlation was used to assess the relationship between marker expression and clinical parameters. Statistically significance was set at *p*-value < 0.05.

## 3. Results

### 3.1. Clinical Data

All consecutive patients who met the inclusion criteria during the study period were enrolled. The study population was composed of 12 males and three females with an average age of 59.86 ± 10.16 years old (range 43–79). As regards exposure to the main risk factors: 2 patients were former smokers and 12 patients were still smokers with consumption of 23.33 ± 8.74 cigarettes per day on average (range 5–40); six patients drank alcohol daily, of which only three were heavy drinkers (≥4 drinks/day). About LPR, the most were not affected (73.34%). In five patients, the serum CRP was found to be higher than 5, which is an indicator of inflammation (Table 1). 

Table 2 shows the characteristics of the LSCC samples in terms of laryngeal subsite involved, pTNM, expression level of PD-L1 and p16 and degree of differentiation. The most patients had glottic (4) or supraglottic (4) or glottic-supraglottic cancer (4), followed by pharyngo-laryngeal (2) SCC. The Combined Positive Score (CPS) was ≥1 in 8 cases and only one biopsy expressed p16 marker. All the enrolled patients underwent surgery as follows: three patients underwent transoral laser microsurgery (TLM), eight patients underwent open partial horizontal laryngectomy (OPHL), and four patients underwent total laryngectomy (TL).

The follow-up (up to 1 October 2024) lasted on average 12.49 ± 7.12 months (range 0.99–23) without recurrence.

### 3.2. Biochemical Data

We investigated a panel of molecular markers involved in oxidative stress regulation and tumor progression to explore their potential clinical significance in LSCC. This analysis included the key proteins SOD, CAT, HO-1, NRF-2, MT, and VIM.

**Figure 1:**
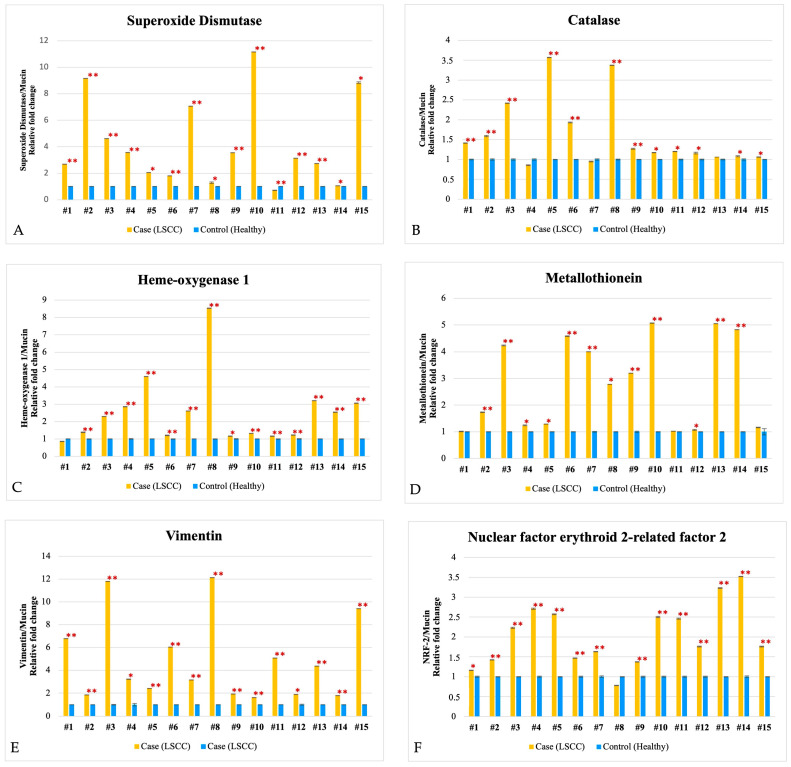
Biochemical markers of oxidative stress and tumor progression in LSCC. Western blotting analysis of (**A**) SOD, (**B**) catalase, (**C**) heme-oxygenase 1, (**D**) metallothionein, (**E**) vimentin, and (**F**) nuclear factor erythroid 2-related factor 2 (NRF2) in LSCC samples (yellow) and adjacent healthy tissues (blue) (*X*-axis). The data represent the densitometric analyses obtained using the Quantity One software. Protein levels were normalized to Mucin (*Y*-axis); the reported values (**A**–**F**), representing the mean of three independent experiments, are expressed relative to healthy tissue (which is set as 1) and the error bars (the black lines above each histogram bar) represent the calculated error of three independent experiments. * *p* < 0.05, ** *p* < 0.01 respect to healthy tissues.

All the evaluated proteins showed significant overexpression in tumor tissues compared to adjacent healthy tissues, as presented in Figure 1. This overexpression highlights the critical role of oxidative stress and related pathways in the pathophysiology of LSCC, as well as the potential contribution of markers associated with tumor progression.

### 3.3. Clinical–Biochemical Correlation

**Figure 2:**
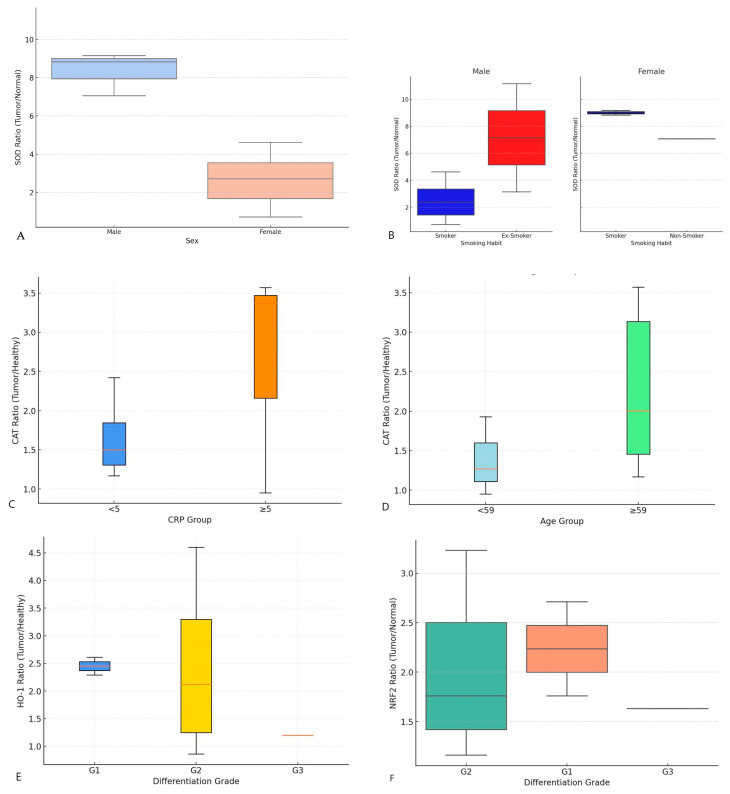
Box plots of biochemical markers in LSCC based on clinical and demographic factors. The box plots show the tumor/normal ratio of protein expression for key oxidative stress-related biomarkers: superoxide dismutase (SOD), catalase (CAT), heme Oxygenase-1 (HO-1), and nuclear factor erythroid 2-related factor 2 (NRF-2) across different patient subgroups. Protein expression levels, normalized to mucin, are presented as ratio of protein expression levels in tumor tissues to that in adjacent healthy tissues. The following trends were observed: (**A**) sex vs. SOD ratio: males show a lower increase in SOD levels compared to females. (**B**) Smoking vs. SOD Ratio: smokers and males show a lower increase in SOD levels compared to non-smokers and females. (**C**) CRP groups vs. CAT ratio: CAT expression is positively associated with C-reactive protein (CRP) levels, with higher ratios observed with higher levels of CRP. (**D**) Age groups vs. CAT ratio: CAT expression is positively associated with age, showing a higher ratio in older patients. (**E**) Differentiation grade vs. HO-1 ratio: HO-1 expression is negatively associated with the degree of differentiation of LSCC, with higher ratios observed in less differentiated tumors. (**F**) Differentiation grade vs. NRF-2 ratio: NRF-2 expression is negatively associated with the degree of differentiation, reducing as differentiation decreases.

Following this observation, we sought to establish possible correlations between the expression levels of these biomarkers and the clinical parameters of the patients enrolled in the study (Table 3). Among the findings, SOD, an enzyme that catalyzes the dismutation of superoxide radicals into hydrogen peroxide and molecular oxygen, showed significant correlations with sex (*p*-value 0.010) and smoking habits (*p*-value 0.013). Interestingly, men and smokers exhibited a lower increase in SOD expression compared to women and non-smokers, respectively (Figure 2A,B). This suggests that SOD activity in LSCC may be influenced by systemic factors such as sex and lifestyle, potentially affecting the tumor’s oxidative environment.

CAT, which detoxifies hydrogen peroxide into water and oxygen, was correlated with CRP levels (*p*-value 0.009), indicating a proportional relationship between systemic inflammation and oxidative stress (Figure 2C). Furthermore, CAT expression was positively associated with age (*p*-value 0.03) (Figure 2D), suggesting that aging contributes to cumulative oxidative damage in tumor tissues.

HO-1, a stress-responsive enzyme involved in heme degradation and cytoprotection, showed significant correlations with tumor differentiation (*p*-value 0.038). Higher HO-1 expression was observed in well-differentiated tumors (G1), whereas poorly differentiated tumors (G3) showed lower levels (Figure 2E). This inverse relationship suggests that HO-1 plays distinct roles depending on the degree of differentiation, potentially reflecting shifts in oxidative stress mechanisms between tumor grades.

NRF-2, a transcription factor that regulates the expression of antioxidant response genes, including HO-1, showed a significant correlation with tumor differentiation (*p*-value 0.03) (Figure 2F). Indeed, NRF-2 levels progressively decrease with increasing tumor de-differentiation, from G1 to G3, reinforcing its role as a driver of defense against oxidative stress in aggressive tumor phenotypes.

MT, known for their ability to scavenge reactive oxygen species and bind heavy metals, did not show statistically significant correlations with clinical data. However, trends nearing significance were observed for laryngeal site involvement and smoking habits, indicating that MT expression may still play a role in certain LSCC subtypes and warrants further investigation.

VIM, a cytoskeletal protein linked to epithelial-to-mesenchymal transition (EMT) and tumor progression, was also overexpressed in LSCC samples. However, no significant correlations with clinical parameters were identified. This lack of association could be attributed to the limited sample size or the possibility that VIM expression reflects more generalized tumor aggressiveness rather than specific clinical or demographic variables.

## 4. Discussion

LSCC represents a significant proportion of HNC globally [2]. The recent literature reports a worsening of the five-year survival in recent decades despite a decrease in incidence and deaths [2,5]. The pathogenesis of LSCC is still little known: studies reported the role of ROS in gene mutations leading to carcinogenesis [17,37]. Indeed, cancer cells, compared to normal cells, tend to have higher levels of ROS. ROS are unstable molecules that can damage DNA, proteins, and other cellular structures. A moderate increase in ROS can stimulate various mechanisms that contribute to tumor development, angiogenesis, metastasis, as well as drug resistance [18,19]. This hypothesis is supported by the fact that the risk factors mentioned above are responsible (directly or indirectly) for oxidative stress [38,39,40,41]. Indeed, smoking can cause both a direct and indirect oxidative effect. In the first case, the direct oxidative effects are caused by free radicals and ROS present in cigarette smoke. Conversely, the indirect oxidative effects are linked to other toxic and carcinogenic components in cigarette smoke, such as aldehyde, and their metabolites, which can provoke a more robust inflammatory response and amplified oxidative damage [42]. So, based on these assumptions, this study highlights the central role of oxidative stress in the pathogenesis of LSCC, focusing on the expression of six key proteins involved in antioxidant defense and tumor biology: SOD, CAT, HO-1, NRF-2, MT, and VIM [43]. The significant overexpression of these proteins in LSCC tissues compared to adjacent healthy tissues underscores the profound oxidative imbalance associated with cancer development. Moreover, the correlations observed between specific proteins and clinical parameters provide valuable insights into their role in LSCC progression.

The observed overexpression of SOD and CAT in tumor tissues reflects an upregulation of cellular mechanisms aimed at mitigating the high levels of ROS found in cancer. Interestingly, SOD expression showed significant correlations with sex and smoking habits, with men and smokers exhibiting a lower increase in SOD expression. This may suggest that chronic oxidative damage, as seen in smokers, overwhelms the capacity of cells to adequately upregulate SOD. The influence of hormonal differences, such as estrogen’s modulation of antioxidant responses, may further explain these findings. There are a few studies focused on SOD activities in LSCC which found a higher SOD activity in cancer tissue than in adjacent cancer-free ones, confirming our results [44,45]. Kalayci et al. found a slightly reduced SOD activity in laryngeal cancer tissues, although this finding was not statistically significant (*p*-value > 0.05) [46]. The authors try to explain this result based on Biri et al.’s hypothesis: they studied prostate cancer and argued that the low SOD activity in cancer may be due to its inability to eliminate high concentrations of ROS [47]. The present study found an overexpression of SOD in the LSCC sample and a statistically significant correlation between sex and smoking habits and an expression ratio of this protein. In particular, the results showed that men and smokers have a lower SOD increase than women and non-smokers, respectively. These data are very interesting considering that smoking is known to cause oxidative stress; however, the small sample size may explain this controversial result. Moreover, it should be kept in mind that men and women have hormonal differences that can affect the expression of antioxidant proteins such as SOD. Estrogen, for example, has been shown to modulate the antioxidant response in different tissues [48,49]. Also, the distribution of habits such as smoking and drinking, which can influence oxidative stress, often varies between men and women [5]. Another hypothesis is that chronic smoking may cause oxidative damage so severe it impairs the ability of cells to produce enough SOD.

Similarly, CAT, another key antioxidant protein, was found to be correlated with systemic inflammation (CRP levels) and age. These results suggest a close interaction between oxidative stress, inflammation, and aging in LSCC pathogenesis, as well as an adaptive response to cumulative oxidative burden over time. Chronic inflammation is known to induce oxidative stress, creating a tumor-promoting microenvironment. Some studies have shown that an elevated inflammatory state, assessed before any treatment, has a negative impact on LSCC prognosis. Specifically, increased CRP levels have been correlated with lower overall survival, greater disease aggressiveness, and a higher risk of post-treatment complications [50,51,52,53]. Additionally, aging is associated with a progressive accumulation of oxidative stress, largely due to mitochondrial dysfunction, impaired antioxidant defense mechanisms, and chronic low-grade inflammation. Our study supports this, as the observed correlation between CAT expression and age suggests that oxidative stress accumulation over time may drive CAT upregulation as a protective response [54]. The only study on CAT activity in LSCC, conducted on 15 smoker patients, found higher enzyme activity in tumor tissues compared to tumor-free adjacent tissues, mirroring our findings. The authors hypothesized that this increased enzyme activity reflects a cellular response to excessive ROS levels in LSCC, reinforcing the role of oxidative stress in tumor biology [44]. Given its role in maintaining redox balance, CAT upregulation in LSCC may contribute to tumor adaptation by neutralizing oxidative stress, thereby supporting cancer cell survival and progression. Understanding this mechanism further could provide insights into potential therapeutic strategies targeting oxidative stress regulation in LSCC.

NRF2 is a transcription factor which, under oxidative stress conditions, dissociates from the Keap1 complex and moves into the nucleus. Once there, it binds to the antioxidant response (ARE) elements present in the promoters of several genes, including HO-1. The activation of HO-1 leads to the degradation of heme into biliverdin, iron, and carbon monoxide, molecules with anti-inflammatory and antioxidant effects. In this way, the NRF2/HO-1 system contributes significantly to cellular protection against oxidative damage and inflammation [4]. In our study, HO-1 and NRF-2 exhibited significant correlations with tumor differentiation. Both proteins followed a similar trend, with HO-1 showing higher expression in well-differentiated tumors (G1), suggesting a protective role in the early stages of LSCC, while NRF-2 levels progressively decreased as tumor differentiation declined. This coordinated downregulation in poorly differentiated tumors (G3) indicates a potential loss of adaptive antioxidant capacity in more aggressive phenotypes, possibly favoring a pro-oxidant environment that drives tumor progression. The interplay between HO-1 and NRF-2 highlights the dynamic regulation of oxidative stress in LSCC and its influence on tumor grade, differentiation, and progression. As mentioned earlier, the production of ROS and antioxidant activity maintains a dynamic balance. High ROS levels contribute to the promotion of carcinogenesis [17,20]. However, when ROS levels rise further, they can become toxic, potentially inducing cell death in cancerous tissues [20,49]. In the current literature, there are only few articles about HO-1 and NRF-2 in LSCC. In 2016, a study conducted in LSCC patients proved that HO-1 protects cell from apoptosis induces by chemotherapeutic agent, such as cisplatin [55]. Another study conducted on serum samples of patients affected by LSCC reported lower HO-1 expression levels in cancer than in health serum samples. These levels were inversed related to cancer stage and neck node involvement: a lower HO-1 expression was shown in the case of advanced tumor and neck node metastasis [56]. On the contrary, in our study, no correlation was found with TNM stage. Regarding NRF-2, the current literature reports that immunohistochemical and Western blot analyses showed that it is only expressed in cancer tissues (in cell nuclei), not in the adjacent health tissues and normal ones [4,57].

MT and VIM provide additional dimensions to the oxidative stress landscape and tumor biology. MT, which plays a role in metal ion homeostasis and ROS neutralization, showed trends of correlation with smoking habits and laryngeal site involvement. These associations, while not statistically significant, suggest that MT may contribute to specific LSCC subtypes. Studies reported higher expression in LSCC samples than in benign laryngeal lesions by immunofluorescence, immunohistochemistry, and Western blot analyses [58,59]. No correlation between MT expression and TNM stage and degree of differentiation was found [54,55]. Ioachim et al. found an overexpression of MT in benign, premalignant, and malignant laryngeal lesions with a statistically significant higher expression in LSCC (*p* < 0.0001) [60]. VIM, a hallmark of EMT, was overexpressed in LSCC tissues, reinforcing its established role in tumor invasiveness and metastasis. Although VIM does not have intrinsic antioxidant properties, it may indirectly interact with antioxidant systems by serving as a scaffold for signaling molecules that modulate ROS levels [61,62,63]. Thus, it may represent an adaptive response to the oxidative stress in the tumor microenvironment. Although no significant correlations were observed with clinical parameters in this study, VIM’s relevance in aggressive cancer phenotypes warrants further investigation. Immunohistochemistry analysis on 69 specimens showed that VIM expression is not related to tumor stage or histological grade. However, VIM-positive LSCC had a worse prognosis with a higher risk of recurrence [64]. Mizdrak et al. evaluated VIM expression in 32 patients affected by LSCC by immunofluorescence. They did not find VIM expression in adjacent health tissue, while its cytoplasmic expression resulted related to the degree of differentiation of LSCC (lower in G1, the highest in G3) (*p* < 0.05). Moreover, VIM expression was higher in case of neck node metastasis, absence of perineural invasion, and supraglottic and trans glottic cancer (*p* < 0.05) [65].

Taken together, these findings illustrate a complex interplay between oxidative stress and tumor progression in LSCC. While SOD, CAT, HO-1, and NRF-2 highlight the intricate regulation of antioxidant defenses, MT and VIM emphasize their broader impact on tumor adaptation and aggressiveness. These results not only provide evidence of oxidative stress as a driving force in LSCC but also suggest potential avenues for the development of diagnostic and prognostic biomarkers.

## 5. Conclusions and Future Perspectives

This preliminary study highlights the significant overexpression of key antioxidant enzymes in LSCC tissues compared to adjacent healthy tissues, suggesting their involvement in the tumor’s adaptive response to oxidative stress. The correlations observed between protein expression levels and clinical parameters—such as SOD with sex and smoking, CAT with CRP and age, and HO-1 and NRF-2 with tumor differentiation—indicate that oxidative stress modulation plays a context-dependent role in LSCC progression.

While antioxidant enzymes generally counteract ROS to maintain cellular homeostasis, their upregulation in LSCC tissues likely reflects a dynamic adaptation to persistent oxidative stress in the tumor microenvironment. Interestingly, the observed progressive downregulation of HO-1 and NRF-2 in poorly differentiated tumors may indicate a loss of adaptive antioxidant responses in more aggressive phenotypes, possibly favoring a pro-oxidant environment that fuels tumor progression.

However, this study is limited by a small sample size, and further large-scale, multicentric studies are required to validate these findings and better define the functional significance of antioxidant enzyme expression in LSCC. Additionally, the use of adjacent healthy tissues as a control may introduce biases due to “field cancerization”, where molecular alterations can already be present in histologically normal tissues. Future research should incorporate functional assays to determine whether the reduction in HO-1 and NRF-2 in aggressive tumors represents an adaptive shift or a vulnerability that could be therapeutically exploited.

From a clinical perspective, understanding how LSCC modulates antioxidant enzyme expression could provide novel insights into biomarker-driven patient stratification and therapeutic decision-making. The identification of specific antioxidant stress-related profiles in LSCC may help to predict tumor aggressiveness, guide treatment selection, and explore the potential for therapies targeting oxidative stress pathways. Furthermore, investigating whether modulating these antioxidant systems enhances the efficacy of conventional treatments, such as chemotherapy, radiotherapy, or immunotherapy, could open new avenues for personalized therapeutic strategies.

Ultimately, a deeper understanding of the role of oxidative stress regulation in LSCC progression may contribute to more refined prognostic tools and treatment approaches, with the potential to improve patient outcomes and quality of life.

## Figures and Tables

**Table 1:** Characteristics of recruited patients.

Characteristics	N (%)
Gender	
Female	3 (20)
Male	12 (80)
Age (years)	
*Mean ± SD*	59.86 ± 10.16
<59 years old	6 (40)
≥59 years old	9 (60)
By gender	
Female (3)	54 ± 12.8
Male (12)	61.33 ± 9.04
*Range*	43–79
Risk factors	
*Tobacco use*	
Non-smoker	1 (6.67)
Light smoker (<20 cigarettes/day)	3 (20)
Heavy smoker (≥20 cigarettes/day)	9 (60)
Former smoker	2 (13.33)
Mean cigarettes/day ± SD	23.33 ± 8.74
*Alcohol abuse*	
Non-drinker	9 (60)
Light drinker (<4 drinks/day)	3 (20)
Heavy drinker (≥4 drinks/day)	3 (20)
*LPR*	
Yes	4 (26.67)
Not	11 (73.33)
Family history of cancer (mother, father, sisters, brothers)	
Yes (lung, breast, prostate, lymphoma, melanoma, gallbladder)	6 (40)
Not	9 (60)
CRP (mg/L)	
Cut-off: <5 mg/L	
<5	10 (66.67)
≥5	5 (33.33)
Total	15 (100)

SD: standard deviation; LPR: laryngopharyngeal reflux; CRP: C-reactive protein.

**Table 2:** Features of laryngeal squamous cell carcinoma.

Parameters	N (%)
Laryngeal involvement	
Supraglottic SCC	4 (26.67)
Glottis SCC	4 (26.67)
Glottic-supraglottic SCC	4 (26.67)
Trans-glottic SCC	1 (6.66)
Pharyngo-laryngeal SCC	2 (13.33)
pT	
Cis	0 (0)
T1	2 (13.33)
T2	9 (60)
T3	0 (0)
T4a	4 (26.67)
pN	
Nx	4 (26.67)
N0	8 (53.33)
N1	1 (6.67)
N2c	1 (6.67)
N3b	1 (6.67)
PD-L1	
CPS < 1	1 (6.67)
CPS ≥ 1	8 (53.33)
CPS ≥ 20	3 (20)
Miss data	3 (20)
+p16	
Negative	10 (66.67)
Equivocal	0 (0)
Positive	1 (6.66)
Miss data	4 (26.67)
Degree of differentiation	
G1	2 (13.33)
G2	9 (60)
G3	1 (6.67)
Miss data	3 (20)
Therapy	
Surgery	
TLM (cordectomies, ESL)	3 (20)
OPHL	8 (53.33)
TL	4 (26.67)
Non-surgical therapy	0 (0)
Adjuvant therapy	
RT	1 (6.67)
CT	0 (0)
CRT	3 (20)
Specimens	
Supraglottis	9 (60)
Glottis	5 (33.33)
Subglottis	1 (6.67)
Total	15 (100)

SCC: squamous cell carcinoma; PD-L1: Programmed Death-1 Ligand 1; CPS: Combined Positive Score; TLM: transoral laser microsurgery; ESL: endoscopic supraglottic laryngectomy; OPHL: open partial horizontal laryngectomy; TL: total laryngectomy; RT: radiotherapy; CT: chemotherapy; CRT: chemoradiotherapy.

**Table 3:** Correlation between biochemical and clinical data by using Pearson’s correlation (*p*-value).

Parameters	SOD Ratio	CAT Ratio	HO-1 Ratio	MT Ratio	VIM Ratio	NRF-2 Ratio
Age	−0.46 (0.086)	0.71 (0.03)	−0.16 (0.69)	0.56 (0.15)	−0.26 (0.42)	−0.38 (0.35)
Sex	−0.64 (0.010)	−0.03 (0.94)	0.28 (0.50)	−023 (0.59)	0.25 (0.43)	−0.26 (0.53)
Smoking	−0.62 (0.013)	−0.43 (0.21)	0.24 (0.57)	−0.70 (0.057)	−0.12 (0.69)	−0.02 (0.92)
Alcohol abuse	−0.30 (0.28)	0.03 (0.93)	0.40 (0.32)	0.33 (0.43)	0.15 (0.63)	0.37 (0.35)
LPR	−0.33 (0.22)	−0.21 (0.58)	−0.25 (0.54)	−0.04 (0.92)	−0.10 (0.74)	−0.69 (0.053)
pTNM	0.13 (0.63)	0.15 (0.68)	0.12 (0.77)	−0.63 (0.09)	−0.36 (0.25)	0.04 (0.86)
Degree of differentiation	0.38 (0.16)	0.18 (0.61)	−0.73 (0.038)	−0.15 (0.73)	−0.28 (0.39)	−0.75 (0.03)
Laryngeal site	−0.26 (0.36)	−0.26 (0.36)	−0.50 (0.20)	−0.63 (0.09)	−0.49 (0.10)	0.23 (0.57)
CRP	−0.27 (0.52)	0.91 (0.001)	−0.17 (0.70)	−0.34 (0.58)	−0.45 (0.16)	0.43 (0.27)
CPS	0.16 (0.71)	0.15 (0.78)	0.32 (0.60)	0.43 (0.47)	0.14 (0.74)	0.92 (0.07)
p16	0.13 (0.63)	N/A	N/A	N/A	−0.31 (0.40)	N/A

SOD: superoxide dismutase; CAT: catalase, HO-1: heme-oxygenase 1; MT: metallothionein; VIM: vimentin; NRF-2: nuclear factor erythroid 2-related factor 2; LPR: laryngopharyngeal reflux; CRP: C-reactive protein; CPS: Combined Positive Score. Boxes in gray if *p*-value < 0.05.

## Data Availability

Data are contained within the article.

## Abbreviations

The following abbreviations are used in this manuscript:

LSCC	Laryngeal Squamous Cell Carcinoma
HNC	Head And Neck Carcinomas
LPR	Laryngopharyngeal Reflux
ROS	Reactive Oxygen Species
AJCC	American Joint Committee on Cancer
SOD	Superoxide Dismutase
CAT	Catalase
TNM	Tumor-Nodes-Metastasis
HPV	Human Papilloma Virus
PD-L1	Programmed Death-1 Ligand 1
CRP	C-reactive protein
CPS	Combined Positive Score
RT	Radiotherapy
CT	Chemotherapy
PBS	Phosphate-Buffered Saline
RIPA	Radioimmunoprecipitation Assay
SDS	Sodium Dodecyl Sulfate
EDTA	Ethylenediaminetetraacetic Acid
PMSF	Phenylmethylsulfonyl Fluoride
HO-1	Heme-Oxygenase 1
MT	Metallothionein
VIM	Vimentin
NRF-2	Nuclear Factor Erythroid 2-Related Factor 2
EMT	Epithelial-to-mesenchymal transition
RT	Room Temperature
SD	Standard Deviation
SEM	Standard Error of the Mean
OPHL	Open Partial Horizontal Laryngectomy
TLM	Transoral Laser Microsurgery
ESL	Endoscopic Supraglottic Laryngectomy
TL	Total Laryngectomy
CRT	Chemoradiotherapy
